# Social orienting and joint attention in preschoolers with autism spectrum disorders

**DOI:** 10.1371/journal.pone.0178859

**Published:** 2017-06-09

**Authors:** Martina Franchini, Bronwyn Glaser, Hilary Wood de Wilde, Edouard Gentaz, Stephan Eliez, Marie Schaer

**Affiliations:** 1 Developmental Imaging and Psychopathology Lab, University of Geneva, Geneva, Switzerland; 2 Department of Psychology and Education (FPSE), University of Geneva, Geneva, Switzerland; 3 Department of Medical Genetics, Geneva University Medical School, Geneva, Switzerland; 4 Stanford Cognitive & Systems Neuroscience Laboratory, Stanford University, Palo Alto, United States of America; Universite de Bretagne Occidentale, FRANCE

## Abstract

Children with Autism Spectrum Disorders (ASD) orient less to socially salient stimuli, such as dynamic social images, than typically developing children. In turn, this lack of social orienting is thought to impair affected individuals’ socio communicative development. Here, we aim to explore the relationship between time spent on dynamic social images and ASD behaviors, such as joint attention and communication, in preschoolers on the autism spectrum. In this study, social orienting is measured using eye-tracking during a task consisting of side-by-side presentations of dynamic social images and dynamic geometric images. The side of the screen where each type of video was presented alternated between items to avoid visual perseveration from influencing the location of participants’ first fixations. Visual exploration patterns recorded during the task from 33 preschoolers with ASD were compared with those of 27 typical developing (TD) children. Additionally, we quantified joint attention behaviors and used standardized parent reports to measure communication. We observed reduced orienting to dynamic social images in preschoolers with ASD compared to TD children. Also, ASD participants went to the dynamic social images less frequently for their first fixations. However, we observed great heterogeneity within the ASD group. ASD preschoolers who spent more time on the dynamic social images also presented more pronounced visual engagement with the dynamic social images (longer mean fixation duration and fewer saccades per second). Moreover, in the ASD group, more time spent on dynamic social images correlated with increased frequency of joint attention behaviors, which in turn correlated with improved communication skills. Our results support reduced social orienting in children with ASD, which correlated with their visual exploration patterns. Further, reduced orienting to the social world in young children with ASD is related to socio communicative deficits and should, therefore, be a focus of intervention programs as early as possible.

## Introduction

The intrinsic ability to orient to our surrounding social environment has been found to be impaired in very young children with autism spectrum disorders (ASD) [1–3]. Furthermore, reduced social orienting in children with ASD has been generally related to behavioral features of ASD (e.g. [4–6]). For this reason, measures of social orienting, or the *“psychological dispositions and biological mechanisms biasing the individual to preferentially orient to the social world”* ([7], p.231), hold promise for improving the early detection of autism. Typically developing (TD) newborns pay special attention to socially relevant cues, such as faces and eyes (e.g., [8]). By contrast, research in the field of autism has demonstrated that young children who go on to be diagnosed with ASD show important differences very early on when responding to socially salient cues. For example, retrospective video analysis demonstrates that compared to their TD counterparts, children who are later diagnosed with autism focus less frequently on people and faces during their early years [9]. This hypothesis is further supported by a study showing that attention to the eyes of a face declines between the ages of two and six months in infants at-risk for developing autism [10].

Reduced attention to the social world may partially explain other social and cognitive features of autism ([11], for a review see 7). When an infant with ASD pays less attention to socially relevant cues, he/she is less socially stimulated and by consequence benefits from less social learning than a typically developing (TD) infant. When a young child orients to a social stimulus during early development by following another person's gaze, it teaches him/her shared attention [12]. These preverbal shared attention experiences, or joint attention (JA) behaviors, represent key precursors for the early socio-communicative development of children with ASD [11]. JA is impaired in young children with ASD [13] and a lack of JA has been described as one of the earliest signs of ASD [14]. JA behaviors can be expressive (initiation of joint attention behaviors, IJA) or receptive (responding to joint attention behaviors, RJA). According to Mundy and his collaborators IJA refers to “*infants’ use of gestures and eye contact to direct others’ attention to objects*, *to events*, *and to themselves*”, and RJA refers to *“infants’ ability to follow the direction of the gaze and gestures of others in order to share a common point of reference*” ([15], p.3). Some studies supporting reduced social orienting in ASD hypothesize that the social world is important for the development of sharing behaviors. As previously explained by Posner [16] and discussed in Mundy and Newell [12], RJA in early development emerges as a consequence of an automatic orientation process toward biologically meaningful stimuli, such as eye and head movements, which underlies shared attention. Also, work by Adamson et al. [17] has shown a positive relationship between social orienting in people and JA engagement in preschoolers with ASD. In summary, ASD development may be partly characterized by a lack of social orienting contributing to decreased social stimulation, and as a result, to diminished occurrences of joint attention behaviors, thereby affecting socio-communication skills. Several studies have demonstrated the effectiveness of eye-tracking for quantifying social orienting. Pierce et al. [6,18] showed reduced orienting towards dynamic social images (DSI, children moving) and increased time spent on dynamic geometric images (DGI, moving geometric shapes or patterns) in toddlers with ASD. Furthermore, time spent on DSI in participants with ASD was positively related to cognitive, language and social skills [6]; whereas individuals with ASD who showed the strongest preference for DGI showed visual patterns of “sticky attention” characterized by a difficulty to disengage from DGI [6,18]. However, the question still remains whether a lack of social orienting in participants could be the result of a “sticky” gaze on one side of the screen, as opposed to an actual preference for one or the other type of stimulus. Because the side of the screen where DSI and DGI videos were presented was not counterbalanced within the task, the paradigm used by Pierce and collaborators [6,18] cannot answer this question. In this study, we were interested in improving our understanding of the relationship between time spent on DSI and JA abilities, and the way in which social orienting and JA are related to sociocommunicative skills in children with ASD. Specifically, we aimed to measure social orienting and then relate it to JA behaviors in a population of young children with ASD. To measure social orienting, we created an eye-tracking task similar to the task used by Pierce and colleagues [6,18]. However, unlike in the original study, we alternated the side of the screen on which the DSI and the DGI appeared to control for participants’ visual attention staying on one side or the other [19,20]. This offered the additional benefit of allowing us to observe the location of the initial fixation, as an additional measurement of automatic orienting to DSI or DGI. We also quantified JA behaviors using the Early Social Communication Scale (ESCS; [21]) and used a standardized measure of adaptive behavior (the Vineland Adaptive Behavior Scales, 2^nd^ edition, VABS-II, [22]) to investigate the relationship between JA behaviors and communication skills. We predicted that social orienting, quantified as the amount of time spent on DSI, would be reduced in our sample of children with ASD compared to TD children, as we already observed in a subgroup of the sample of children with ASD included in the present study [23]. We additionally predicted that the first fixation on DSI would also be reduced in our sample of children with ASD compared to TD children. Second, we postulated that social orienting would positively correlate with the amount of JA behaviors in toddlers with ASD. Finally, we predicted that social orienting and JA behaviors would positively correlate with communication skills.

## Materials and methods

### Participants

#### ASD participants

A total of 36 participants (31 males) with ASD were included in the study. Three participants with ASD (all males) were excluded from analyses and from participant description because of lack of quality in data collection. Participants ranged in age from 19 to 51 months (see Table 1). Participants with ASD were recruited through French-speaking parent associations and specialized clinical centers. The University of Geneva Institutional Review Board approved the study protocol for all participants and participants’ parents gave their individual written informed consent prior to inclusion in the study. To confirm previously established clinical diagnoses, we used either the Autism Diagnosis Observation Schedule–Generic (ADOS-G) [24] or the Autism Diagnosis Observation Schedule, second edition (ADOS-2), which includes a toddler module [25]. To allow for comparisons between scores from different modules, we applied Gotham et al.’s algorithm to the scores [26]. Of the 27 participants evaluated with the ADOS-G (Module 1 or 2), 21 children met criteria for ASD and 6 for Autism. The mean severity score of the participants was 7.36 (S.D. = 1.77) out of 10 according to the transformation [26]. Six other children were evaluated using the ADOS-2 (toddler module). At time of evaluation, five fell in the moderate to severe range of concern and one fell in the mild to moderate range.

**Table 1:** Demographics, adaptive functioning and features of joint attention behavior in the ASD and TD groups.

**Demographics**			
	**ASD (n = 33)**	**TD (n = 27)**	**Significance**
**Age (months)**	33.7 (S.D. = 8.7)	30.8 (S.D. = 11.6)	*t* = 1.11, *p* = 0.271, *df* = 58
**Gender (n)**	Males = 28 /Females = 5	Males = 15 /Females = 12	*x*^2^ *= 6*.*28*, *p = 0*.012*, *df* = 58
**Adaptive functioning—Vineland Adaptive Behavior Scales, 2**^**nd**^ **edition (Sparrow et al., 2005)**			
	**ASD (n = 32)**	**TD (n = 27)**	**Significance**
**Adaptive behavior composite (SS)**	74.9 (S.D. = 9.9)	101.2 (S.D. = 7.2)	*t* = 11.44, *p*<0.001***, *df* = 57
**Communication (SS)**	72.7 (S.D. = 12.5)	104.7 (S.D. = 8.8)	*t* = 11.21, *p*<0.001***, *df* = 57
**Daily living skills (SS)**	78.5 (S.D. = 11.5)	101.7 (S.D. = 8.8)	*t* = 8.60, *p*<0.001***, *df* = 57
**Socialization (SS)**	75.3 (S.D. = 9.9)	100.2 (S.D. = 6.4)	*t* = 11.24, *p*<0.001***, *df* = 57
**Motor skills (SS)**	85.3 (S.D. = 12.8)	99.4 (S.D. = 8.3)	*t* = 4.92, *p*<0.001***, *df* = 57
**Joint attention behaviors—Early Social Communication Scale (Mundy et al., 2003)**			
	**ASD (n = 25)**	**TD (n = 23)**	**Significance**
**Initiating Joint Attention (n)**	5.8 (S.D. = 5.8)	23.3 (S.D. = 2.1)	*t* = 7.35, *p*<0.001***, *df* = 46
**Responses to Joint Attention (n)**	6.4 (S.D. = 4.7)	11.7 (S.D. = 0.6)	*t* = 4.52, *p*<0.001**, *df* = 46

* indicates a *p*-value <0.05.

** indicates a *p*-value <0.01.

*** indicates a *p*-value <0.001.

ADOS assessments were administered and scored by psychologists who had met the requirements for research reliability. Before they were included in our research protocol, most participants had already been diagnosed with an ASD. The toddlers (younger than 30 months of age), who were assessed using the Toddler Module of the ADOS-2, all had been labeled as “high concern” by a trained clinician in a specialized clinical center in Geneva (the CCSA) before being referred to the study.

### TD participants

Twenty-seven TD children (15 males) aged 14 to 57 months participated in the study. Before their inclusion in our research protocol, all children were screened for neurological/psychiatric problems and learning disabilities through an interview over the phone and a medical developmental history questionnaire completed before their initial visit. All TD participants were assessed using the ADOS-G. The mean severity score from their assessments was 1.04 (S.D. = 0.19) and we had no clinical concerns for them. TD participants were recruited through announcements in the Geneva community. As with the ASD participants, parents gave their informed consent prior to inclusion in the study. There was no difference in age between the ASD and TD groups, but gender distribution was significantly different (see Table 1).

#### Description of participants’ adaptive functioning and joint attention skills

The children with ASD scored lower on adaptive functioning (as measured by the Vineland Adaptive Behavior Scales, 2^nd^ edition [22]) compared to the TD group. TD participants all scored in the “adequate” range for their adaptive level when using the instrument’s normative group. Six preschoolers with ASD obtained an adaptive level of “adequate”, 17 were in the “moderately low” range and nine of them fell in the “low” range. The two groups also differed on their Communication, Daily Living Skills, Socialization and Motor Skills domain scores. Their performance on the Early Social-Communication Scale [21] revealed impaired joint attention in the children with autism as a group compared the TD children. See Table 1 for a summary.

### Assessment instruments

#### Time spent on dynamic social images

**Fig 1:**
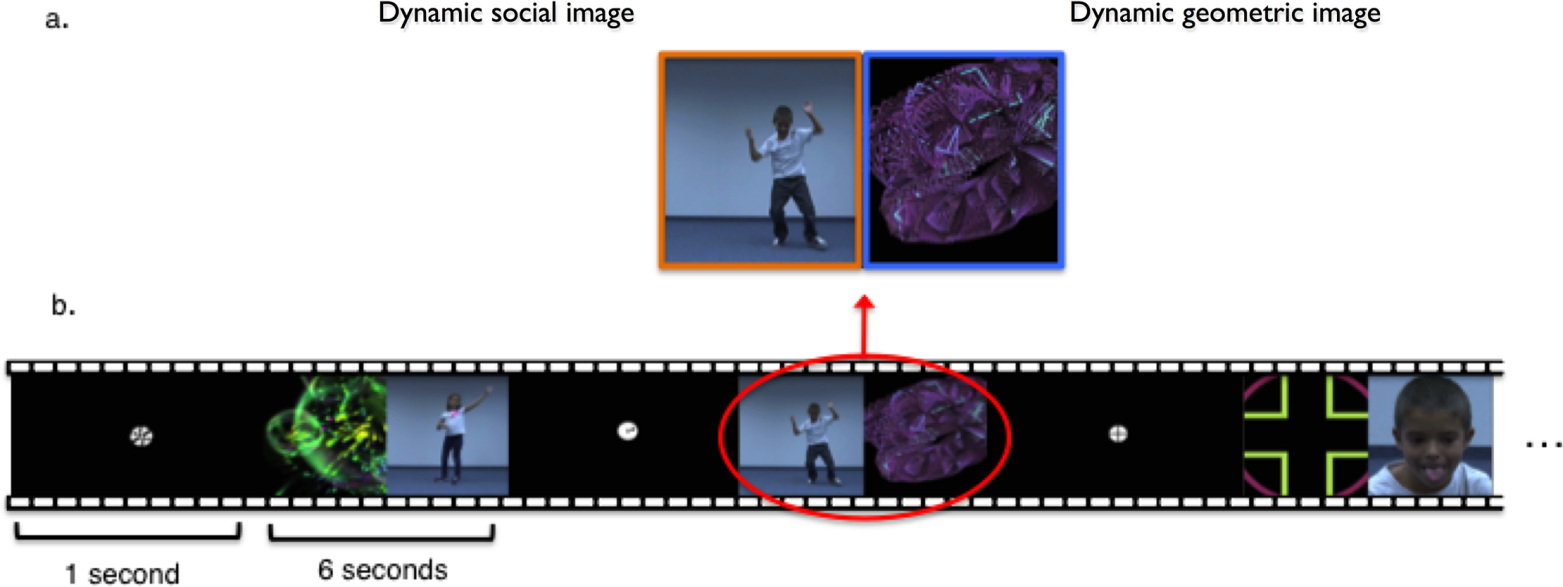
a. Screenshot representing the DSI-TASK task (adapted from Franchini et al., 2016, [23]). Areas Of Interest (AOI) correspond to the left (DSI, in orange) and the right (DGI, in blue) halves of the screen. b. The DSI-TASK task is composed of ten discrete segments. DSI and DGI stimuli are randomly assigned to the left or right sides of the screen for each segment. Between each segment, we used a turning wheel to bring participants’ visual attention to the center of the screen.

We designed a Dynamic Social Images task (DSI-TASK), inspired from the task proposed by Pierce et al. [18], that we recently described in another paper [23] and in which 16 participants with ASD and 9 TD children from the present study were included. This passive 1-minute task consists of simultaneous presentations of dynamic geometric images (DGI) and dynamic social images (DSI) on either side of the screen of an eye-tracking machine. For the DGI, we used moving geometrical shapes that are similar to the classic abstract screen savers. These images were either taken from Mac OS screensavers or from http://www.reallyslick.com/screensavers, where they are available to the general public. DSI were sequences of children moving and dancing solo (Fig 1). The DSI stimuli in Pierce et al. [18] were videos of children doing yoga outside. To differentiate participants’ attention to the children from their interest in background details (e.g. wind in the leaves tree, flowers etc.), we filmed our stimuli in an indoor, neutral environment with a white background. Our DSI-TASK task had additional differences from Pierce et al.’s task: 1) We used ten 6-second video segments, as opposed to one 60-second block. 2) Within the segments, the presentation sides for DGI and DSI were counterbalanced between the left and right sides of the screen. 3) Between each 6-second segment we added a turning wheel, placed in the middle of the screen on a black background, to bring participants’ attention back to the center of the screen. These last two differences enabled us to analyze the location of participants’ initial saccades (the first point where a person fixes his/her attention).

The task was administered using Tobii Studio software (www.tobii.com) with T60XL and TX300 Tobii eye-trackers. The sampling rate was 60Hz for the T60XL and 300Hz for the TX300, but the video resolution and screen size were identical for both devices (1920x1080, 17-inch displays). The Tobii machine can tolerate moderate head movement up to 60cm from the screen. For the analyses, we used Tobii Studio software, version 3.1.6. A I-VT filter was enabled during analysis. (Classifier: 30°/s; Velocity calculator window length: 20ms). The merge fixations option was further enabled (Max. time between fixations: 75ms; Max angle between fixations: 0.5°). Following data collection, Areas of Interest (AOI) were drawn (Fig 1) on the videos to delineate stimulus type (DSI (in orange) and DGI (in blue)). For each kind of stimuli (DSI and DGI), we calculated several variables: the total fixation duration (total summed looking time), the mean duration of each fixation (fixation length), and the number of saccades per second (movement of the eye between fixation points). As an additional measure for automatic orienting to social stimuli, we calculated the percentage of segments for each participant, when the first fixation was on the DSI. This percentage was calculated by dividing the number of first fixations on DSI by the number of the segments (10). Children sat on their parents’ laps, 60cm from the screen. Before administering the task, all participants completed a five-point calibration procedure adapted to toddlers to verify accurate and complete eye motion and eye gaze detection. The procedure consisted of following an image of a pet animal on the screen with both eyes. Calibration was repeated until each participant hit all five points with both eyes.

#### Joint attention behaviors

To measure joint attention (JA), we used the Early Social-Communication Scale [21]. This play-based instrument allowed us to quantify IJA (amount of spontaneous eye contact, as well as pointing and showing behaviors for shared attention) and RJA (participants’ ability to follow the examiner’s proximal and distal pointing gestures). The sessions are recorded and the IJA and RJA behaviors are subsequently coded according to the manual. Successful RJA is quantified during 14 examiner-prompted opportunities. The ESCS also accounts for IJA events spontaneously initiated by the child, during this 20-minutes evaluation. The sessions are video-recorded and the IJA and RJA behaviors are subsequently coded according to a manual. Each video was double-coded and discussed until the raters reached consensus. Eight of the 33 participants with ASD in our sample didn’t finish the ESCS because the sitting time was too long for them or because they became disengaged, and thus stopped concentrating. Given that scoring is based on the frequency of behaviors during the entire task, we excluded the eight participants from ESCS analyses. For the TD group, we were able to use data for 23 out of 27 participants.

#### Communication assessment

We used the Vineland Adaptive Behavior Scales, 2^nd^ edition [22] to measure communication. The VABS-II is a standardized parent interview for the assessment of adaptive functioning. We used the VABS-II interview to obtain standard scores (SS) for communication, and for three other adaptive behavior domains: Daily Living, Socialization and Motor Skills. The VABS-II also produces a SS for an adaptive behavior composite. We conducted the entire VABS-II for all participants in the study.

### Statistical analyses

Participants who looked at the screen for less than 50% of the DSI-TASK were excluded from the analyses (three boys with ASD). Visual exploration on the DSI-TASK was operationalized in the following ways: 1) percentage of fixation time spent on each stimulus type (DSI and DGI) was calculated by dividing the number of fixations on each half of the screen (by stimulus type) by the total number fixations on the entire screen; 2) the percentage of first fixation on DSI was calculated by dividing the number of segments during which the first fixation was on the DSI image by the total number of segments (10); 3) fixation length was measured by calculating the mean fixation duration for each kind of stimulus (DSI and DGI); 4) the number of saccades per second was calculated by dividing the total number of saccades by the total fixation time on each stimulus type. Distribution normality was tested with the D’Agostino-Pearson omnibus normality test. Student’s t-test or Mann-Whitney tests were conducted in PRISM 6.0 for Mac OS X with diagnosis as a fixed variable. Next, we used correlation analysis (Pearson or Spearman according to the distribution) to test for a relationship between joint attention (on the ESCS), mean fixation duration or the number of saccades per second and the percentage of time spent on DSI. Finally, we used correlation analyses to test the relationship between joint attention (from the ESCS) and communication (SS from the VABS-II). Finally, we report some exploratory analyses correlating the percentage of time spent on DSI with the adaptive domains from the VABS-II. Results were considered significant at *p*≤0.05. However, as the VABS-II includes 4 domains, an alpha level of *p*≤0.0125 was used for analyses involving the VABS-II domains. Correlations involving IJA and RJA scores from the ESCS were considered significant at an alpha level of *p*≤0.025.

## Results

### Time spent on DSI is reduced in children with ASD

**Fig 2:**
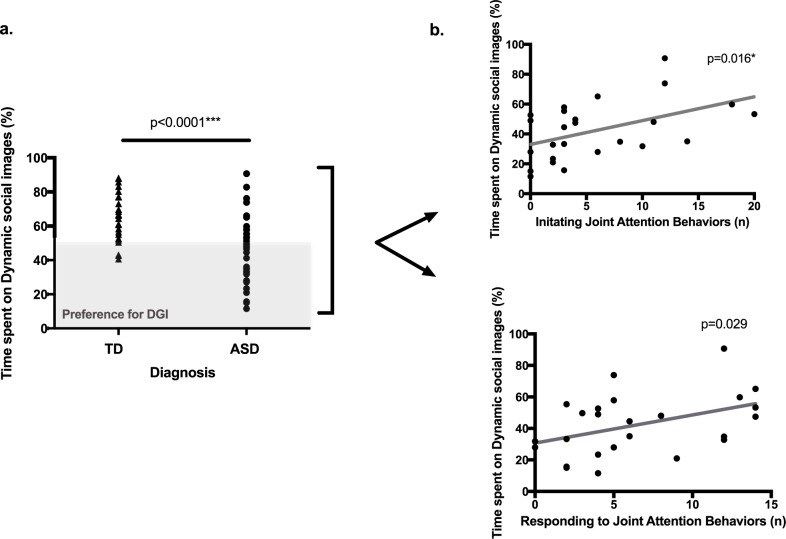
a. Differences in percentage of time spent on DSI for the DSI-TASK task between the ASD and TD groups. Participants represented in the grey zone preferred DGI. b. Correlations for each group between time spent on DSI during the DSI-TASK and IJA and RJA from the ESCS.

The total time spent looking at the screen was not statistically different between the ASD and TD groups (*U* = 395, *p* = 0.458; median time in the ASD group = 48.82 sec., range: 34.07–59.97; median time in the TD group = 52.69 sec., range: 30.00–59.59). The percentage of time spent on DSI was 45.0% (S.D. = 19.7) in the ASD group, which means that, on average, children with autism spent more time (55.0%) looking at DGI. Among the 33 youngsters with autism, 21 of them (63.6%) spent more time on DGI (i.e. looked at DGI more than 50% of the time). By contrast, the TD group looked more at DSI. Accordingly, they spent 65.2% (S.D. = 14.1) of their total time on DSI. Out of 27 TD children, only three (11.11%) looked more at DGI. The difference in the fixation duration on DSI between the two groups is statistically different (*t* = 4.48, *p*<0.001, *df* = 58). Results are summarized in Fig 2. Lastly, correlational analyses showed that there was no effect of age on time spent on DSI in either the ASD (*r* = -0.20, *p* = 0.256, *df* = 31) or the TD (*r* = -0.33, *p* = 0.088, *df* = 25) groups.

### TD participants look initially at DSI more than children with ASD

The TD group went to DSI more frequently for their first fixations (69.6% of the times, S.D. = 12.9) than the ASD group (53.9% of the times, S.D. = 24.7), a difference that is statistically significant (*t* = 2.98, *p* = 0.004, *df* = 58). These results are summarized in Table 2.

**Table 2:** Between-group comparisons on fixation duration and first fixation to DSI.

	ASD	TD	Significance
**Fixation Duration, % (S.D.)**	45.0 (19.7)	65.2 (14.1)	t = 4.48, *p*<0.001***, *df* = 58
**First Fixation, % (S.D.)**	53.9 (24.7)	69.6 (12.9)	t = 2.98, *p* = 0.004**, *df* = 58

* indicates a *p*-value <0.05.

** indicates a *p*-value <0.01.

*** indicates a *p*-value <0.001.

### Within the ASD group, time spent on DSI correlates with visual exploration patterns

To explore if the amount of time spent on DSI was correlated with exploration patterns, we analyzed the mean fixation duration and the average number of saccades per second on DSI for the ASD and the TD groups. The ASD group demonstrated shorter fixations on DSI stimuli than the TD group (Average fixation length on DSI, ASD: 0.37 sec., S.D. = 0.02, TD: 0.43 sec., S.D. = 0.01; *t* = 2.11, *p* = 0.042, *df* = 58). When we analyzed number of saccades, no difference in saccades was found between the ASD and the TD groups for DSI (saccades on DSI in the ASD group: median = 1.95 n/sec., range: 0.59–4.76; saccades on DSI in the TD group: median = 2.03 n/sec., range: 0.73–5.97; *U* = 426, p = 0.779, *df* = 58). In the ASD group, our results indicate a positive correlation between Time spent on DSI and Fixation duration on DSI (r = 0.45, p = 0.008) and a negative correlation between Time spent on DSI and the number of saccades on DSI (r = -0.40, p = 0.018). In the TD group, we did observe a significant correlation between time spent on DSI and either mean fixation duration (r = 0.06, p = 0.784) or the number of saccades per second (r = 0.09, p = 0.651).

### Time spent on DSI is positively correlated with JA behaviors

In the ASD group and according to multiple comparisons correction, time spent on DSI was positively correlated with IJA on the ESCS (*r* = 0.48, *p* = 0.016, *df* = 23). The significance of the correlation between the time spent on DSI and RJA was close to the Bonferroni-corrected cut-off significance threshold (*r* = 0.43, *p* = 0.029 *df* = 23), (see Fig 2). In the group of TD children, there were no correlation between the occurrences of IJA or RJA and time spent on DSI (*r* = -0.24, *p* = 0.262, *df* = 21; *r* = -0.09, *p* = 0.691, *df* = 21).

### Amount of JA behaviors in ASD is positively correlated with communication

**Fig 3:**
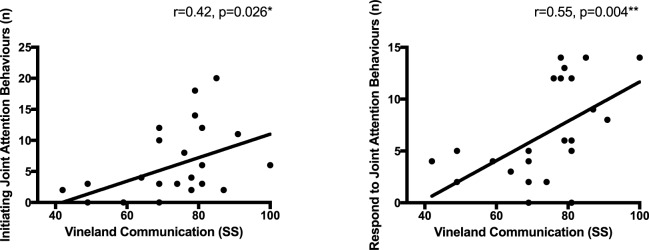
Relationship between JA behaviors and communication measure within the ASD group.

As shown in Fig 3, the quantity of IJA (*r* = 0.42, *p* = 0.026, *df* = 23) and the quantity of RJA (*r* = 0.55, *p* = 0.004, *df* = 23) behaviors correlated with VABS-II Communication SS in the ASD group. In the TD group, neither the occurrences of IJA *(r* = -0.24, *p* = 0.262, *df* = 20) or RJA (*r* = -0.03, *p* = 0.903, *df* = 20) correlated with VABS-II Communication SS.

### Time spent on DSI and correlation with adaptive scores on the VABS-II

We additionally examined the relationship in the ASD group between time spent on the DSI and the SS obtained from the VABS-II (the total adaptive behaviors composite and the 4 domains). There was a positive correlation between time spent on DSI and the adaptive behavior composite (*r* = 0.43, *p* = 0.012, *df* = 30). Regarding the adaptive behavior domains from the VABS-II, there was a significant positive correlation between time spent on DSI and the Socialization SS (*r* = 0.43, *p* = 0.012 *df* = 30), and between time spent on DSI and the Communication SS (*r* = 0.48, *p* = 0.004, *df* = 30). However, we did not observe significant relationships between DSI and the Daily Living Skills SS (*r* = 0.27, *p* = 0.123, *df* = 30) or the Motor Skills SS (*r* = 0.35, *p* = 0.046, *df* = 30)

### Gender effects

Given that the gender ratio in our sample was not equally distributed (Table 1), we checked our results in the previous sections while using only the male participants from the ASD and TD groups. All results presented in the previous sections remained significant in male-only samples. Additionally, we decided to compare males and females from the TD group to explore any gender effects on the main results on the DSI-TASK task. The results showed no gender differences for total fixation duration (*t* = 1.67, *p* = 0.107, *df* = 25) or first fixation on DSI (*t* = 1.40, *p* = 0.175, *df* = 25).

## Discussion

In this study, we observed reduced orienting through dynamic social images (DSI) in young children with ASD compared to typically developing (TD) children, though the ASD group showed pronounced heterogeneity. Moreover, our ASD participants oriented less frequently to DSI with their first fixations than TD children. Correlations between time spent on DSI and visual exploration patterns (mean fixation duration and number of saccades per second) showed that children with ASD who spent more time on DSI also showed increased visual engagement with DSI (longer fixations and lesser saccades). Within the ASD group, time spent on DSI was positively correlated with the amount of joint attention behaviors (JA). Lastly, joint attention correlated with the Communication score from the VABS-II in our group of young children with autism.

### Reduced social orienting in ASD

In the current study, we replicate Pierce et al.’s finding that orienting to DSI is reduced in preschoolers with ASD [18]. The fact that we were able to observe similar results using a different sample and a slightly modified paradigm provides clear evidence for reduced social orienting in autism [1,2,7,11]. Moreover, the similarity between our results and those reported by Pierce and collaborators [18] also adds support for eye-tracking technology as a valid method for measuring social orienting. The current study further demonstrates that children with ASD initially orient less frequently to DSI than their TD peers (53.9% of the times in ASD and 69.7% of the times in TD children), suggesting that automatic orienting to social information might be reduced in preschoolers with ASD. Children with ASD who spent more time on DSI showed greater visual engagement (longer fixations) with DSI. Furthermore, children with ASD who spent more time on DSI also showed more advanced verbal and non-verbal communication skills. This finding may be helpful for understanding both reported differences in time spent on social stimuli [27,28] among young children on the autism spectrum, as well as poor engagement with the social environment in autism [29]. Pierce and colleagues [6,18] observed disparate visual exploration patterns between the group of children with ASD who preferred the DGI and the one who preferred DSI. Participants with a preference for DGI demonstrated fewer saccades on DGI, which fits the pattern for enhanced visual attention as well as a certain difficulty disengaging from DGI. Their observations are supported by Elsabbagh and colleagues [30], who have suggested that “sticky” gaze may be an early sign of ASD. Our correlation analyses show that children with autism who spend less time on DSI show less visual engagement (shorter mean fixation duration and more saccades per second). These results support the idea that children with autism with superior social orienting are likely to demonstrate greater visual engagement when observing a social scene.

### Relationships between social orienting, joint attention and communication

Our results suggested relationships between time spent on DSI, quantity of JA behaviors and socio-communicative learning. Visual orienting to DSI is automatic as well as integral to the development of JA [16,31]. Indeed, disengaging one’s attention from social stimuli during the first year of life has been shown to be a predictor of an ASD diagnosis later in life [32]. There is a logical connection between a child’s attention to his social environment and his ability to learn from it [15]. In our study, we observed that social orienting in preschoolers with ASD is positively related to the amount of JA behaviors that they exhibit. In other words, the more a child orients to social stimuli, the more joint attention behaviors we can expect him/her to express ([11], for a review see 7). JA represents the sharing of attention and experience, both of which are necessary for learning social cognition skills [33]. When JA behaviors are reduced, as in ASD, learning opportunities are also reduced, with dramatic consequences for young children’s social and, at times, cognitive development. Mundy et al. [34] support this idea by demonstrating relationships between JA, measures of IQ and social competence in young children with autism. The putative contributions to social and communicative development from initiating JA behaviors (IJA) and responding to JA behaviors (RJA) in ASD is an ongoing discussion [35]; however, numerous studies have shown that both IJA and RJA are related to language development [14,36,37]. In the current study, social orienting is positively correlated with both IJA and show a clear tendency toward significance with RJA. Our results also suggest better communication in children with stronger JA skills. Further analysis of the connection between social orienting and adaptive measures highlight a relationship between social orienting and adaptive functioning. These results, consistent with those of Pierce et al. [6], suggest that children with ASD who prefer DSI perform better in social and communication domains than children who prefer DGI. Indeed, communication and social skills are often intertwined when it comes to preschool development [38,39]. Furthermore, daily living skills have been positively related to communication [40], and non-verbal communication in particular [41]. These data lead us to postulate that if social orienting improves communication, it may increase adaptive functioning as well.

### Limitations and perspectives

The present study has several limitations. 1) The percentage of females in our sample, which is at 15.2%, does not represent the percentage observed in the general ASD population. The Centers for Disease Control and Prevention (CDC, 2014) estimated females as between 19.6% and 27.8% of 8-year-old children with ASD. Interestingly, a recent study by Kemp [42] shows that females with ASD are diagnosed later than boys on average, which may explain the smaller proportion of females in our preschool-aged sample. Even if little is known about the role gender differences play in time spent on and the processing of DSI in ASD, neuroimaging studies suggest possible gender differences in the cerebral processing of DSI in healthy adults [43,44]. 2) Our sample size was relatively small. These results should be explored in a larger sample size. Moreover, there a large number of participants were unable to finish the ESCS in the ASD group, which further reduced our ability to complete our correlation analyses. 3) Our study didn’t include a comparison group of children with development delay (DD), which would allow for developmental-age matching with the ASD group. In our study, the group of children with ASD and the TD children showed differences in their general abilities. Hence, this difference could be, at least in part, the reason of the differences in visual preference observed between the two groups. A DD control group allows for relevant comparisons and discussions about the specificity of social and communication disorders in ASD compared to other developmental disorders. This point could have been addressed by collecting standardized developmental measures, an element that was, unfortunately, missing from our protocol. However, similar studies by Pierce et al. that include a DD control group [6,18], suggest that a preference for DGI is specific to ASD. 4) Even if the current study points to a relationship between social orienting and communication in preschoolers with autism, our cross-sectional design does not allow us to draw conclusions about causality, which needs to be addressed through a longitudinal study. 5) The measures used in our sample gave us three types of data: eye-tracking data, JA behaviors, and parent report. Although the results showed a strong relationship between the different types of data, the difference in the way they were collected could have introduced some bias (e.g. eye-tracking assessment is accurate within the evaluation context, whereas parent-reports are more generic). That said, the measures included in the current study are thought to be sensitive to the assessment of ASD behaviors. Lastly, given the discrepant findings for the social orienting hypothesis in high-risk infants [10], we assume that social orienting and its implications for social-communicative development would be more accurately addressed in children at-risk for ASD during the first months of life. Indeed, early diagnosis of autism spectrum disorder (ASD) remains a challenging yet important objective [45,46]. By identifying and describing symptoms of ASD as early as possible and by implementing high quality interventions, we can dramatically improve developmental outcomes [47–49]. This point is further supported by a published paper from our team, partially including in part the same participants, which demonstrates that time spent on DSI predicts a better clinical outcome after one year [23]. As suggested by Pierce et al. [6], there is a pressing need to develop and validate tools, such as eye-tracking paradigms assessing time spent on DSI, that can help clinicians to diagnosis autism as early and as accurately as possible.

## Conclusions

Eye-tracking paradigms measuring time spent on dynamic social images and dynamic geometrical images represent promising tools for measuring social orienting in toddlers with ASD. Social orienting and joint attention are impaired in preschoolers with ASD, and thus may be key to finding ways to improve early diagnosis of ASD. Furthermore, reduced orienting to the social world in autism appears to be related to social-communicative impairment, making it an important objective for young participants benefitting from intervention programs (e.g. in interventions based on social engagement [47]).
